# Median arcuate ligament syndrome: a cost analysis to determine the economic burden of a rarely diagnosed disease

**DOI:** 10.3389/fpsyg.2023.1166744

**Published:** 2024-01-16

**Authors:** Christopher L. Skelly, Colleen Stiles-Shields, Hayley Goldenthal, Nicole Bohr, Estee Feldman, Grace Zee Mak, Tina Drossos

**Affiliations:** ^1^Department of Surgery, Section of Vascular and Endovascular Surgery, University of Chicago Medicine, Chicago, IL, United States; ^2^Institute for Juvenile Research, Department of Psychiatry, The University of Illinois at Chicago, Chicago, IL, United States; ^3^Department of Psychiatry and Behavioral Sciences, University of Chicago Medicine, Chicago, IL, United States; ^4^Department of Nursing Research, University of Chicago Medicine, Chicago, IL, United States; ^5^Department of Surgery, Section of Pediatric Surgery, University of Chicago Medicine, Chicago, IL, United States

**Keywords:** median arcuate ligament syndrome, chronic abdominal pain, economic burden, cost analysis, rare disease, cognitive behavioral therapy, coping

## Abstract

**Background:**

Chronic abdominal pain (CAP) is a medical condition resulting in enormous economic burden and healthcare utilization costs. One understudied source of CAP is the median arcuate ligament syndrome (MALS). MALS is often not diagnosed and treated for a variety of reasons, including the fact that MALS is highly comorbid with psychological symptoms and psychiatric disorders similar to CAP. To better inform future work on the study of MALS, we undertook a pilot study to estimate the economic impact and public health burden of this condition. We hypothesized that MALS imposes a significant public health burden.

**Methods:**

Pediatric and adult patients enrolled in a prospective study undergoing multidisciplinary evaluation and treatment for MALS at a tertiary care facility were invited to participate in a brief self-report survey, the Direct and Indirect Medical Care Impact of MALS Form, to capture health care resources including procedures, surgeries, health care visits, and absenteeism (school and work). To estimate costs from the Direct and Indirect Medical Care Impact of MALS Form, the medical care usage data self-reported by patients were converted to dollar value utilizing FSC-93 billing data and corresponding current procedural terminology (CPT) codes for procedures and provider visits one year prior to surgery and then following surgery. Descriptive analyses were conducted to characterize the sample in terms of demographics and reported absences from school and work.

**Results:**

One hundred and nineteen patients (mean age = 30.9 ± 13.0) completed the questionnaires, yielding a 57% response rate. 82.4% (*n* = 98) of the participants were female and 90.8% (*n* = 108) were non-Hispanic/Latine white. The mean and median surgical follow-up periods were 5.3 and 5.4 years, respectively. Overall, median cost of provider and ancillary healthcare provider visits for each patient was (US)$19,119 including the pre-operative and post-operative visits. The mean cost for providers alone was (US)$28,908. Wilcoxon signed-ranks tests indicated that the postoperative missed number of days of school were significantly lower than the pre-surgical number of missed school days (Z = −3.36, *p* = 0.001). Similarly, there were significantly less missed work-days following surgery than before for the entire sample (Z = −2.86, *p* = 0.004).

**Conclusion:**

Median arcuate ligament syndrome imposes a large economic burden on patients and the healthcare system. The current findings, although reflective of a homogenous population, are adding to a growing body of literature suggesting that healthcare disparities play a role in the low rates of diagnosis and treatment of MALS.

## Introduction

Chronic abdominal pain (CAP) is considered one of the most common presenting concerns in inpatient and outpatient hospital visits and affects approximately half of all adults during their lifetime (Lakhoo et al., 2021). Among pediatric populations, CAP is similarly impactful and accounts for 2–4% of all primary care visits (Starfield et al., 1980). CAP impacts multiple domains of functioning and is associated with reduced quality of life, negative psychological outcomes (e.g., anxiety, depression, low self-efficacy), functional impairment, and loss of productivity (e.g., missed school and work; Ramchandani et al., 2007; Shelby et al., 2013). Encompassing a broad range of differential diagnoses, CAP often requires an extensive medical workup to arrive at a diagnosis and appropriate treatment plan. Prior work demonstrates that patients with MALS have similar symptoms and psychosocial profiles to those with CAP (Mak et al., 2016). The associated indirect and direct costs of CAP, especially for those with unknown etiology, amount to enormous economic burden and healthcare utilization costs (Yarger and Sandberg, 2020). As an example, abdominal pain is the leading GI cause of patients seeking doctor’s visits (Peery et al., 2022) and the direct and indirect cost of Irritable Bowel Syndrome have been reported to be as high as US$8 billion and $25billion, respectively, (Ashburn and Gupta, 2006). Unfortunately, economic data on rare diseases is scarce (Angelis et al., 2015).

One understudied source of CAP is median arcuate ligament syndrome (MALS, also referred to as the celiac compression syndrome, or Dunbar Syndrome). MALS is a vascular compression syndrome that results in chronic, reproducible epigastric abdominal pain, nausea, and weight loss and has radiographic evidence of celiac artery compression (Harjola, 1963). Although there are surgical procedure to relieve the compression, symptoms attributable to the compression are improved in only 70–80% of patients (Reilly et al., 1985; van Petersen et al., 2009; Tulloch et al., 2010; El-Hayek et al., 2013; Mak, 2017; DeCarlo et al., 2023). These suboptimal surgical outcomes reflect the poorly understood pathophysiology of the pain from the compression and result in many surgeons not offering the operation (Szilagyi et al., 1972). This reluctance to operate results in a barrier to patients. MALS is a lifespan condition, such that it is well documented to be diagnosed in pediatric, adult, and older adult patients. Celiac artery compression as determined by autopsy and radiographic studies is estimated to occur in approximately 3–33% of the total population (Szilagyi et al., 1972; Ilica et al., 2007), suggesting that 9.9–23.2 million individuals in the United States may be impacted by MALS. Although many individuals with radiographic features of celiac artery compression are asymptomatic (Szilagyi et al., 1972; Park et al., 2001; Ilica et al., 2007), there remains a large number of symptomatic patients without typical features found on CT scan (Patel et al., 2019) who are undiagnosed and/or do not receive appropriate treatment for their symptoms (Rezigh et al., 2015).

Multiple systemic and condition-driven factors interplay to make accurate diagnosis and treatment of MALS difficult. First, MALS is a diagnosis of exclusion, requiring patients to undergo an extensive diagnostic workup to rule out other sources of abdominal pain (Mak et al., 2013; Skelly et al., 2018; Skelly and Mak, 2021). This process has been reported by former patients and their families as necessitating high levels of resources, ability to travel and miss school/work, and frequent advocacy for the full workup to occur (Stiles-Shields et al., 2021, 2022). Second, there is a lack of knowledge and awareness about MALS among the general population and medical community (Mak et al., 2016), decreasing the likelihood of screening for the condition in the face of CAP. As such, MALS is both underdiagnosed and frequently misdiagnosed as chronic functional abdominal pain (CFAP; Mak et al., 2016). Further, given the highly homogenous demographic characteristics of patients, misdiagnosis likely occurs at higher rates for patients with minoritized identities—who are already less likely to receive proper diagnosis and treatments for pain conditions (Mossey, 2011; Johnson et al., 2013; Goyal et al., 2015). Third, MALS is highly comorbid with psychological symptoms and psychiatric disorders (Mak et al., 2016). These comorbidities can distract from accurate diagnosis (e.g., providers focusing on anxiety symptoms for pain etiology) (Stiles-Shields et al., 2021); as well as create barriers to seeking and/or maintaining care (Skelly and Mak, 2023). Further, surgical intervention for MALS does not appear to resolve psychological symptoms and psychiatric disorders for pediatric or adult patients (Skelly et al., 2018; Stiles-Shields et al., 2018). The persistence of psychological impacts may lead some providers to question the success of surgical intervention, despite consistent findings for surgery significantly reducing pain and increasing quality of life (Mak et al., 2013; Joyce et al., 2014; Skelly et al., 2018). Finally, even when diagnosed, patients with MALS are unlikely to complete surgical release. Indeed, in the United States between 1999 and 2011, only 3% of adult patients diagnosed with MALS underwent surgical release (Rezigh et al., 2015). In sum, MALS stands as a condition that leads to significant impairments and burdens (Joyce et al., 2014; Mak et al., 2016; Skelly et al., 2018), yet due to a variety of factors is overlooked, misdiagnosed, and undertreated.

To better inform education, outreach, assessment, intervention, and follow-up methodologies for these patients, it is imperative that the public health burden of this condition is fully elucidated. As such, the goal of the current study was to conduct a pilot evaluation of the economic impact of MALS. Total costs accrued at the site of the surgical release was considered as a means to characterize costs. However, the difficulties described in receiving an accurate diagnosis (Stiles-Shields et al., 2021) necessitate a broader approach to cost evaluation, as multiple disciplines and medical systems are typically engaged around diagnosis and treatment for MALS. Therefore, to achieve this aim, pediatric and adult patients who underwent surgical intervention for MALS were assessed via self-report for their pre- and post-surgical medical services and costs. Grounded within the framework of the Consolidated Health Economic Evaluation Reporting Standards (CHEERS; Husereau et al., 2013), we hypothesized that MALS imposes a significant public health burden, such that patients miss work, school, and accrue considerable costs for medical care. To date, this is the first study documenting the financial impact of MALS.

## Methods

### Participants

All patients with a diagnosis of MALS and who underwent evaluation and treatment at the University of Chicago Medicine were enrolled in an Institutional Review Board (IRB) approved prospective study that has previously been described (Mak et al., 2016; Skelly et al., 2018; Stiles-Shields et al., 2018). To the best of our knowledge, the University of Chicago Hospitals MALS program is the only interdisciplinary, lifespan focused, MALS clinical research site that draws national and international patients, making it an ideal site to begin to evaluate the economic burdens of diagnosis and treatment of MALS. Inclusion for the purpose of this study were all consented patients from the prospective study of any age, who completed surgical release of the median arcuate ligament at the University of Chicago Medicine (i.e., not all participants enrolled in the prospective study underwent surgery). All who met these criteria were invited to participate, with the exception of those who had difficulty reading, speaking, or understanding English, as the study materials were in English.

### Procedure

In compliance with the University of Chicago Institutional Review Board (IRB), digital informed consent was obtained from all participants (Stiles-Shields et al., 2021). For participants under the age of 18, a parent or legal guardian was asked to provide guardian consent in addition to the minor patient’s assent. All former patients meeting the inclusion criteria were sent an email invitation to complete the digital informed consent and survey. Participants were compensated with (US)$10 upon return of survey data. Survey data were collected and managed using REDCap electronic data capture tools hosted at the University of Chicago (Harris et al., 2009). Participants were compensated for survey completion.

### Measures

#### Demographics

All participants were asked to report the following information: age, sex, current medical diagnosis (es), race/ethnicity, current living situation (e.g., with parents or independently), and current work/school attendance.

#### Health care impact

The Direct and Indirect Medical Care Impact of MALS Form (Supplementary data) is a survey developed by the University of Chicago MALS team (Stiles-Shields et al., 2021) and adapted from an investigation of the cost of irritable bowel syndrome and functional abdominal syndrome (Hoekman et al., 2015). This survey assessed health care utilization, travel associated with seeking medical care, and missed days from work or school 1 year prior to surgery and since surgery. The 1 year time frame was selected based on standard post-operative follow up and to provide equal time frames pre and post-surgery for comparison. Patients and parents were asked about frequency and types of out-patient hospital consultations and procedural episodes.

### Data analysis

Descriptive analyses were conducted to characterize the sample in terms of demographics and reported absences from school and work. To estimate direct costs from the Direct and Indirect Medical Care Impact of MALS Form, the medical care usage data self-reported by patients were converted to dollar value utilizing FSC-93 billing data and corresponding current procedural terminology (CPT) codes for procedures and provider visits 1 year prior to surgery and then following surgery. Totals were calculated by multiplying the costs by number of visits, consistent with previous healthcare cost research (Hoekman et al., 2015). Using SAS 9.4 (SAS Institute, Cary NC), pre and post-operative missed days of school and work were examined using Wilcoxon signed-ranks tests.

## Results

### Participants

In aggregate, 207 adults and children with celiac artery compression and chronic abdominal pain who underwent laparoscopic release of the median arcuate ligament at a single tertiary care center for MALS between December 2010 and April 2019 were invited to participate in an online survey. The survey was administered between November and December 2019. Following the removal of participants with missing data and parents of pediatric patients who participated without their children, 119 participants were included in the current study, yielding a 57% response rate. The sample ranged in age from 13 to 70 (mean age = 30.9 ± 13.0). The majority of the sample was female (82.4%) and white (90.8%). Table 1 displays the sample demographics. The mean and median surgical follow-up periods were 5.3 and 5.4 years, respectively.

**Table 1:** Sample characteristics, median (SD).

Age	30.91 (13.01)
**Gender, *n* (%)**	
Male	21 (17.6%)
Female	98 (82.4%)
Non-binary/gender identity not listed	0 (0%)
**Ethnicity, *n* (%)**	
Hispanic/Latino/a/x	5 (4.2%)
Not Hispanic/Latino/a/x	114 (95.8%)
Race, *n* (%)^*^	
American Indian or Alaska Native	2 (1.7%)
Asian	3 (2.5%)
Black/African American	3 (2.5%)
White	108 (90.8%)
More Than One Race	3 (2.5%)
Household Income (US$)	$110,983.70 ($216,465.04)

^*^Race and ethnicity are reported in a way that is consistent with the United States of America’s National Institutes of Health (2023).

### Medical utilization costs

Patients reported that they sought care from multiple health care providers, undergoing significant numbers of tests and procedures. Table 2 displays the appointment costs and utilization pre- and post-operatively and Table 3 displays procedural costs. The overall median total cost for the treatment and care of a patient with MALS was (US)$19,119 but ranged from $2,388 to $207,557; this includes appointment costs where the pre-operative median cost was $4,110, and the post-operative median cost was $3,756. This translates to a significant number of appointments, emergency room visits, hospital days and procedures performed. Pre-operatively, the mean number of annual medical visits was 14 visits per patient; post-operatively, the annual number of visits was 13 per patient (Table 2). There was a demonstrable shift from patients seeing specialists, school doctors, and dieticians in the pre-operative period, to psychologists and physical therapists in the post-operative period. Patients continued to see their primary physician at equal rates pre- and post-operatively. Post-operatively, procedure volumes dropped in the overall number of patients undergoing procedures as well as the median number of procedures performed per patient (Table 3). This corresponded to a reduction in procedural costs per patient.

**Table 2:** Annual direct medical appointment costs for patients undergoing surgery for MALS, median (IQR).

	Pre-surgery				Post-surgery			
	Patients (*N*)	Appointments per patient	Appointments total (range)	Appointment cost	Patients (*N*)	Appointments per patient	Appointments total (range)	Appointment cost
**Appointment provider**								
Primary care	72	5.5 (3.0, 10.0)	1–100	$990 (540, 1800)	71	5.0 (3.0, 8.0)	1–108	$900 (540, 1,440)
Specialist	75	6.0 (4.0, 12.0)	1–100	$2,772 (1848, 5,544)	59	8.0 (3.0, 20.0)	1–216	$3,696 (1,386, 9,240)
School MD/RN	13	5.5 (2.0, 10.0)	1–100	$2,140 (778, 3,890)	5	6.0 (2.0, 12.0)	1–12	$2,334 (778, 4,668)
Psychiatrist	18	3.0 (1.0, 6.0)	1–22	$858 (286, 1716)	15	5.0 (2.0, 24.0)	1–60	$1,430 (572, 6,864)
Psychologist	23	4.0 (1.0, 9.0)	1–25	$1,156 (289, 2,601)	25	8.0 (3.0, 15.0)	1–260	$2,312 (867, 4,335)
Social worker	6	3.0 (1.0, 5.0)	1–52	$867 (289, 1,445)	3	25.0 (6.0, 60.0)	6–60	$7,225 (1879, 17,340)
Physical therapy^*^	6	4.0 (1.0, 24.0)	1–100	–	13	23.5 (15.0, 30.0)	1–40	–
Dietician	23	2.0 (1.0, 3.0)	1–12	$348 (174, 522)	13	2.0 (1.0, 3.0)	1–20	$348 (174, 522)
Total	84	14.0 (8.0, 35.0)	2–210	$4,128 (2,413, 10,684)	84	13.0 (5.0, 40.0)	1–345	$3,753 (1,272, 11,991)

^*^Cost data not available for physical therapy appointments; IQR, interquartile range; MALS, median arcuate ligament syndrome.

**Table 3:** Annual procedural costs for patients undergoing surgery for MALS, median (IQR).

	Pre-surgery				Post-surgery			
	Patients (*N*)	Appointments per patient	Appointments total (range)	Appointment cost	Patients (*N*)	Appointments per patient	Appointments total (range)	Appointment cost
**Procedure type**								
Nerve block	20	1.0 (1.0, 1.0)	1–4	$605 (605, 605)	20	1.0 (1.0, 2.8)	1–14	$605 (605, 1815)
SCS placement	0	–	–	–	3	1.0 (1.0, 3.0)	1–3	$2,566 (2,566, 7,698)
Upper GI endoscopy	70	1.0 (1.0, 2.0)	1–6	$882 (882, 1,323)	31	1.0 (1.0, 3.0)	1–8	$882 (882, 1764)
Endoscopic ultrasound	22	1.3 (1.0, 2.0)	1–10	$2075 (1,383, 2,766)	11	2.0 (1.0, 3.0)	1–20	$2,766 (1,383, 4,149)
Colonoscopy	49	1.0 (1.0, 1.0)	1–10	$1,228 (1,228, 1,228)	19	1.0 (1.0, 2.0)	1–3	$1,228 (1,228, 2,456)
Laparotomy	1	–	1	$5,474	0	–	–	–
Laparoscopy	6	1.0 (1.0, 1.0)	1–1	$2,427 (2,427, 2,427)	3	1.0 (1.0, 2.0)	1–2	$2,427 (2,427, 4,854)
Angiogram	12	1.0 (1.0, 2.0)	1–2	$1786 (1786, 3,572)	13	1.0 (1.0, 1.0)	1–1	$1786 (1786, 1786)
Angioplasty/stenting	0	–	–	–	6	1.0 (1.0, 1.0)	1–2	$2,196 (2,196, 2,196)
Vascular ultrasound	56	2.0 (1.0, 2.0)	1–10	$2068	20	2.0 (1.0, 2.5)	1–18	$2068 (1,034, 2,585)
Total	81	4.0 (2.0, 5.0)	1–27	$3,984 (2,110, 5,736)	53	2.0 (1.0, 6.0)	1–52	$2,417 (1,228, 6,886)

^*^IQR, interquartile range; MALS, median arcuate ligament syndrome; SCS, spinal cord stimulator; GI, gastrointestinal.

### Missed work and school

In addition to the number of appointments, patients reported missing significant time from both work and school. Table 4 displays the self-reported, pre-surgical and postoperative time missed from work or school for the full sample and by age group: children (<22; *n* = 71), young adults (22–34; *n* = 44), and adults (>35; *n* = 36). Self-reported postoperative missed days of work was limited to those who reported missing 100 days or less due to an extreme outlier for the adult group (i.e., reporting missing 513 days of work since surgery). Wilcoxon signed-ranks tests indicated that the postoperative missed number of days of school were significantly lower than the pre-surgical number of missed school days (Z = −3.36, *p* = 0.001). Similarly, there were significantly less missed work days following surgery than before for the entire sample (Z = −2.86, *p* = 0.004). There was no evidence of significant differences based on age group for these categories, with the exception of young adults missing significantly fewer school days following surgery (Z = −3.42, *p* = 0.001).

**Table 4:** Self-reported missed school and work days prior to and following surgery, median (IQR).

	Pediatric (<22; *n* = 71)		Young adult (22–34; *n* = 44)		Adult (35–65; *n* = 36)		Full sample (*n* = 151)	
**Missed school days**								
Pre-surgery	22.50 (24.75)	Z = −0.85, *p* = 0.4	5.00 (30.00)	Z = −3.42, *p* = 0.001	0.00 (0.00)	Z = −1.07, *p* = 0.3	5.00 (25.00)	Z = −3.36, *p* = 0.001
Post-surgery	5.00 (32.50)		0.00 (4.00)		0.00 (0.00)		0.00 (4.00)	
**Missed work days**								
Pre-surgery	0.00 (11.75)	Z = −0.85, *p* = 0.4	0.00 (10.00)	Z = −1.55, *p* = 0.1	10.00 (300.00)	Z = −1.88, *p* = 0.06	2.00 (11.00)	Z = −2.86, *p* = 0.004
Post-surgery	0.00 (4.50)		0.00 (5.00)		0.00 (1.00)		0.00 (5.00)	

## Discussion

Grounded in the framework of CHEERS (Husereau et al., 2013), the current study was the first of its kind to pilot an economic evaluation for the diagnosis and intervention of pediatric and adult MALS. Consistent with previous findings (Stiles-Shields et al., 2021), patients with MALS who completed surgery reported attending a high number of healthcare appointments, across a variety of specialties. Median costs of care associated with MALS diagnosis and treatment totaled (US)$19,119. Types of care sought from specialty providers shifted in discipline: from specialty care associated with physical symptoms of MALS prior to surgery to post-surgical care seeming to be more highly associated with comorbid conditions. Finally, missed school and work days significantly improved following surgical release.

The current findings implicate MALS as creating a significant public health burden in line with previously established economic burdens and healthcare use costs associated with CAP (Yarger and Sandberg, 2020). Yet, compared with some other causes of CAP, MALS has the potential to be more insidious in its impacts. This increase is due to the previously described factors that lead to the condition being overlooked, misdiagnosed, and undertreated (Rezigh et al., 2015; Mak et al., 2016). Indeed, as previously noted through qualitative data from patients with MALS, additional specialty provider appointments and diagnostic procedures are common to simply receive an appropriate diagnosis (Stiles-Shields et al., 2021). The current study extends the previous findings, identifying the resultant median healthcare cost of (US)$19,119 accrued from commonly performed diagnostic and surgically-related procedures (e.g., upper GI endoscopy, vascular ultrasound) and repeated appointments with primary care providers and specialists. Further, indirect costs, such as missed work and school across these procedures and appointments, were also commonly reported. Such lost productivity contributes to substantial societal costs (Gannon et al., 2013). Similar to other GI conditions, MALS is a lifespan condition that occurs in pediatric and adult patients; given the low mortality in MALS and low intervention rate (Rezigh et al., 2015), there is the risk of compounding prevalence such that direct and indirect costs have the potential to adversely impact patients, caregivers, and society exponentially with time (Kaplan, 2015).

The burden identified by the diagnosis and intervention of MALS also highlights likely healthcare disparities associated with this condition. Participants in the current study reported an average household income of US$110,983. This amount far exceeds the typical US household income based on recent census data (US$70,784; Income in the United States: 2021, 2022), yet is still impactful with the median overall healthcare costs for MALS identified in this study (US$19,119). As such, the current sample likely had: (1) the health insurance coverage, (2) above average financial resources, and (3) flexibility in work and/or childcare, to attend appointments and procedures–and manage the associated costs. Notwithstanding the clear absence of minoritized racial and ethnic identities in previous research of patients with MALS (Mak et al., 2013; Joyce et al., 2014; Skelly et al., 2018; Stiles-Shields et al., 2021, 2022), the socioeconomic status of patients who are able to receive a diagnosis and treatment is likely higher than average. Critically, the presence of privileged experiences and identities repeatedly identified in patients with MALS is a call to action to increase equitable healthcare for patients with CAP.

While this study primarily focused on the economic impact of MALS, the findings also implicate psychosocial needs of this patient population. Following surgical intervention, when pain symptoms typically decrease and quality of life improves (Mangione et al., 1997), participants reported increased use of healthcare by mental health supports (e.g., psychologist, social worker). Much like other conditions that cause CAP, MALS is highly comorbid with psychological symptoms and psychiatric disorders (Mak et al., 2016). While surgery improves pain symptoms and quality of life, it has not been associated with improved psychological symptoms for pediatric or adult patients (Skelly et al., 2018; Stiles-Shields et al., 2018). As such, comorbid psychological symptoms and/or psychiatric disorders could be appropriately identified and targeted with empirically-supported interventions, such as cognitive behavioral therapy (CBT), prior to surgical intervention. Similarly, to reduce the burden associated with MALS across the diagnostic and intervention experience, patients would likely benefit from an interdisciplinary assessment and treatment approach, including Primary Care, GI, Surgery, Pain, Radiology, as well as Psychiatry. Such responsive screening and intervention pre-operatively may reduce the number of specialists and appointments required post-operatively and improve long-term quality of life.

The current study had multiple strengths, including being the first of its kind to examine direct and indirect costs associated with the diagnosis and treatment of MALS across pediatric and adult patients. However, the findings should also be considered in light of specific limitations. First, visit and procedure totals were self-reported, which leaves room for ample error. While it would have been ideal to have had these totals extracted from patient charts, patients with MALS typically access care across multiple medical centers and health systems, particularly patients who live in more rural locations or places that typically do not treat MALS. As such, extracting totals from the surgical intervention site would have omitted much of the care many patients received. Second, the sample from the current study all received surgical intervention through the care of an interdisciplinary team at a single medical center located in the Midwestern United States. It is unclear how these findings might extend to other regions or medical centers treating MALS. Third, the sample was highly homogenous in terms of reported socioeconomic status, gender, racial/ethnic identities, and survey response rates. While this is reflective of previous samples of patients with MALS, it points to the critical need to engage in more inclusive recruitment practices, starting with broader education for providers, screening, and intervention for potential patients with MALS. Such engagement could certainly also begin to address nonresponse bias in this study as well.

The current study was grounded in the CHEERS framework (Husereau et al., 2013) and piloted an economic evaluation of the diagnostic and intervention costs for pediatric and adult MALS. Participants reported significant healthcare utilization, resulting in median costs of (US)$19,119. Multiple procedures and specialty care appointments were reported, with type of specialty care typically shifting from primarily medical preoperatively to primarily psychological care post-operatively. Findings implicate a significant public health burden associated with MALS, as well as the need to expand the equitable screening and care for this condition, as well as increased interdisciplinary treatment for pediatric and adult patients with MALS.

## Data availability statement

The raw data supporting the conclusions of this article will be made available by the authors, without undue reservation.

## Ethics statement

The studies involving humans were approved by University of Chicago Institutional Review Board (IRB). The studies were conducted in accordance with the local legislation and institutional requirements. Written informed consent for participation in this study was provided by the participants’ legal guardians/next of kin.

## Author contributions

CS, CS-S, and TD conceptualized and designed the study. All authors contributed to the article and approved the submitted version.
