# Supplementary material for: Median arcuate ligament syndrome: a cost analysis to determine the economic burden of a rarely diagnosed disease
Source: Front Psychol. 2024 Jan 16;14:1166744. doi: 10.3389/fpsyg.2023.1166744 (PMC10824967; doi:10.3389/fpsyg.2023.1166744)
Supplement: Supplementary file 1 [file Table_1.DOCX]

**Survey of Direct and Indirect Medical Care Impact of MALS**

Date of Surgery: ­­­­­_________________

For the following questions, please answer if you (or your child) had any of the following experiences in the one year prior to surgery and/or since you (or your child) have had surgery:

|  | One Year Prior to Surgery | | | Since Surgery | | |
| --- | --- | --- | --- | --- | --- | --- |
|  | No | Yes | # of Times | No | Yes | # of Times |
| **Medical Appointment:** |  |  |  |  |  |  |
| Pediatrician/Primary Care Physician |  |  |  |  |  |  |
| Other Medical Specialist |  |  |  |  |  |  |
| School Doctor/Nurse |  |  |  |  |  |  |
| Psychiatrist |  |  |  |  |  |  |
| Psychologist |  |  |  |  |  |  |
| Social Worker |  |  |  |  |  |  |
| Physical Therapist |  |  |  |  |  |  |
| Dietician |  |  |  |  |  |  |
| Other |  |  |  |  |  |  |
| **Hospital Care:** |  |  |  |  |  |  |
| Emergency Room/Department |  |  |  |  |  |  |
| MALS Surgery *(if you have had more than one, please note in “Since Surgery” Columns)* | - |  | *(Please indicate # of days in hospital)* |  |  | *(Please indicate # of days in hospital)* |
| Other Hospital Admission |  |  | *(Please indicate # of days in hospital)* |  |  | *(Please indicate # of days in hospital)* |
| **Medical Procedures:** |  |  |  |  |  |  |
| Nerve Block |  |  |  |  |  |  |
| Spinal Cord Stimulator |  |  |  |  |  |  |
| Upper GI Endoscopy (EGD) |  |  |  |  |  |  |
| Endoscopic Ultrasound |  |  |  |  |  |  |
| Colonoscopy |  |  |  |  |  |  |
| Laprotomy |  |  |  |  |  |  |
| Laparoscopy |  |  |  |  |  |  |
| Angiogram |  |  |  |  |  |  |
| Angioplasty/Stent |  |  |  |  |  |  |
| Vascular Ultrasound |  |  |  |  |  |  |
| Cholecystectomy |  |  |  |  |  |  |
| **Surgical Procedures** |  |  |  |  |  |  |
| Other Surgeries (i.e. appendectomy) |  |  |  |  |  |  |
| **School/Work:** |  |  |  |  |  |  |
| Did you miss days of school due to the symptoms, diagnosis, and/or treatment of MALS? |  |  |  |  |  |  |
| Did you miss days of work due to the symptoms, diagnosis, and/or treatment of MALS? |  |  |  |  |  |  |
| **Parent/Guardian**: Did you miss days of work/school due to the symptoms, diagnosis, and/or treatment of your child’s MALS? |  |  |  |  |  |  |

**Medication**

Please list your current medication names and dosage:

| **Medication** | **Dosage** |
| --- | --- |
|  |  |
|  |  |
|  |  |
|  |  |
|  |  |
|  |  |
|  |  |
|  |  |
|  |  |
|  |  |

**Travel**

1. How many times did you travel to the University of Chicago Medical Center in the one year before your/your child’s surgery: ____
2. How many times did you travel to the University of Chicago Medical Center since your/your child’s surgery: ____
3. How did you typically travel to the University of Chicago Medical Center?
   1. Personal car
   2. Taxi/Uber/Lyft
   3. Bus/Train
   4. Airplane
   5. Other: ________

*If the University of Chicago Medical Center was not your primary medical care location, please answer items 4 through 8; otherwise, please skip to the next section.*

1. Approximate distance from your home to the medical center where you/your child received the most care in the one year prior to surgery: _______ miles
2. How many times did you travel to the medical center where you/your child received the most care in the one year prior to surgery: ____
3. Approximate distance from your home to the medical center where you/your child received the most care since surgery: _______ miles
4. How many times did you travel to the medical center where you/your child received the most care since surgery: ____
5. How did you typically travel to the medical center where you/your child received the most care?
   1. Personal car
   2. Taxi/Uber/Lyft
   3. Bus/Train
   4. Airplane
   5. Other: ________
